# Cross‐Cultural Applicability and Application of the Nursing Leaders’ Readiness for Artificial Intelligence Scale: A Cross‐Sectional Study

**DOI:** 10.1155/jonm/9637045

**Published:** 2026-07-29

**Authors:** Hui Yang, Yuanzhi Guo, Yaxin Qiao, Weihui Bai, Cancan Chen, Hengyu Hu

**Affiliations:** ^1^ Nursing Department, Henan Provincial People’s Hospital, Henan Provincial Intelligent Nursing and Transformation Engineering Research Center, Zhengzhou University People’s Hospital, Henan University People’s Hospital, Zhengzhou Henan, 450003, China, hnsrmyy.net; ^2^ Department of Medical Oncology, Henan Provincial People’s Hospital, Henan Provincial Intelligent Nursing and Transformation Engineering Research Center, Zhengzhou University People’s Hospital, Henan University People’s Hospital, Zhengzhou Henan, 450003, China, hnsrmyy.net; ^3^ Department of Thoracic Surgery, Henan Provincial People’s Hospital, Henan Provincial Intelligent Nursing and Transformation Engineering Research Center, Zhengzhou University People’s Hospital, Henan University People’s Hospital, Zhengzhou Henan, 450003, China, hnsrmyy.net

**Keywords:** a cross-sectional study, cross-cultural applicability, nursing leader, readiness for artificial intelligence

## Abstract

**Aims:**

To translate, revise and evaluate the Chinese‐version Nursing Leaders’ Readiness for Artificial Intelligence Scale and assess Chinese nursing leaders’ AI readiness.

**Design:**

Cross‐sectional survey.

**Methods:**

The research team conducted translation, cognitive interviews, a pilot survey and psychometric evaluation. Survey participants were 762 nursing managers. The reliability and validity of the Chinese‐version scale were examined. The status and influencing factors of AI readiness among Chinese nursing managers were investigated.

**Results:**

The translated scale comprises 20 items. Cronbach’s α was 0.824. Regarding validity, the three‐factor model demonstrated a good fit. The square roots of the average variance extracted, absolute values of correlation coefficients among dimensions and content validity index values were acceptable. The mean AI readiness score was 65.27 ± 9.78. Of the participants, 624 scored above 60 points, 136 scored 40–60 points and 2 scored below 40 points. Scores differed significantly by hospital type, hospital level, department, training participation, prior AI tool or system use and frequency of AI usage.

**Conclusion:**

The Chinese‐version scale demonstrated good reliability and validity and can effectively assess AI readiness among Chinese nursing managers. Nursing leaders generally demonstrated a favourable level of AI readiness; however, factors such as hospital type and level remain significant determinants influencing nursing leaders’ AI readiness.

**Implications for Nursing Management:**

This study provides a standard measurement tool for Chinese hospitals to check how ready their nursing managers are for AI. These findings help us understand the current state of AI readiness among nursing managers in China. The results can help improve AI use strategies. In addition, they can help bring AI into nursing practice more smoothly. This study has important value for moving forward with digital change and smart nursing in China.

## 1. Introduction

Artificial intelligence (AI) is profoundly transforming healthcare service delivery. In nursing practice, its applications span multiple domains, including clinical decision support systems, patient outcome predictive analytics and intelligent nursing documentation [1, 2]. Moreover, AI tools have extended beyond administrative functions to practical nursing education and clinical skill assessment. For instance, Erkayıran [3] demonstrated that an AI‐based system could assess nursing students’ therapeutic communication skills within a standardised patient‐based training programme with accuracy and reliability comparable to those of human raters, while also offering greater objectivity and scalability. This evidence suggests that AI integration in nursing has moved from theoretical potential towards practical application, highlighting the need for strategic leadership at the organisational level [3]. However, the successful integration of AI in nursing practice largely depends on organisational readiness, particularly the preparedness of nursing managers, who are key drivers of technology adoption. Research has indicated that leadership support is among the most critical factors determining the success or failure of health information technology adoption in clinical settings [4]. Without adequate managerial preparedness, even the most advanced AI systems may fail to achieve their intended benefits [5].

Kotp et al. developed the Nursing Leaders’ Readiness for AI Integration scale and conducted a survey among nursing leaders from nine private hospitals in Cairo, finding that more than one‐third of the participants demonstrated a high level of AI integration readiness. We conducted a cross‐cultural validation of this assessment tool in China and assessed the AI readiness of nursing leaders in Chinese public hospitals.

## 2. Background

### 2.1. Advancement of AI in the Nursing Field

AI has become a transformative force in the healthcare sector and is rapidly expanding across multiple domains, including clinical practice, management and education [6]. Driven by breakthroughs in machine learning algorithms, enhanced computational capabilities and the exponential accumulation of digital health data, the global healthcare AI market has experienced unprecedented growth. The World Health Organization [7] has recognised AI as key for achieving universal health coverage and issued guidance on the ethical deployment of AI technologies in the health sector.

In nursing practice, AI technology provides significant opportunities to enhance nursing care quality and improve patient outcomes. Current applications span multiple domains, including clinical decision support systems that assist nurses in identifying changes in patient conditions, predictive analytics for adverse events such as falls and pressure injuries, natural language processing technologies for nursing documentation and intelligent systems for resource allocation and workforce management [8]. These technologies have demonstrated the potential to reduce nurses’ cognitive burden, minimise errors and enable timely clinical intervention.

Despite the broad application prospects of AI, its successful integration into nursing practice poses numerous challenges. Research indicates that most AI healthcare applications remain in the development or pilot phase, with limited translation into routine clinical practice [9]. Implementation barriers include technological infrastructure limitations, data quality issues, workflow integration challenges and, critically, the readiness of healthcare professionals and organisations to adopt these technologies [5].

### 2.2. Core Concepts of AI Readiness

AI readiness refers to the state of preparedness of organisations or individuals to adopt and effectively utilise AI technologies and encompasses multiple dimensions, including knowledge, skills, attitudes, resources and infrastructure [10]. In the medical field, AI readiness refers to the ability and willingness of healthcare organisations and workers to adopt AI technologies. These technologies are mainly applied in three areas: supporting doctors in clinical diagnosis and treatment decision‐making, assisting patients in achieving better therapeutic outcomes and enhancing the operational and administrative efficiency of hospitals [11].

The implementation of AI depends not only on the maturity of the technology itself but also on systematic preparedness at both the organisational and personnel levels. The complexity of nursing work, humanistic nature of nurse–patient interactions and context‐dependent characteristics of nursing decision‐making pose unique challenges for AI applications in the nursing domain [8]. Therefore, AI readiness assessment is particularly important in the nursing field.

### 2.3. Importance of AI Readiness for Nursing Leaders

Nursing managers assume important responsibilities, including driving changes in nursing practice, facilitating the adoption of new technologies and cultivating nursing talent [12]. Research indicates that leadership support is a critical factor in determining the successful implementation of health information technology [13]. As leaders of nursing teams, nursing managers’ perceptions of and attitudes towards AI technology influence subordinate nursing staff through multiple pathways. First, nursing managers’ positive attitudes can foster a supportive organisational climate for AI adoption, thereby reducing resistance to new technologies among nursing staff. Second, nursing managers have decision‐making authority in areas such as resource allocation, training arrangements and workflow adjustments, which enables them to provide the necessary organisational support for AI implementation. Third, through role‐modelling and daily communication, nursing managers can effectively convey the value and benefits of AI to enhance nursing staff’s acceptance of it [14].

Compared with clinical nursing staff, nursing managers must understand AI technology from a more macro perspective, not only mastering the basic principles and application scenarios of AI but also having the capability to assess the feasibility of AI projects, manage AI implementation risks and measure the return on AI investment [15]. Assessing AI readiness can provide a basis for nursing managers to develop AI implementation strategies, thereby helping healthcare institutions appropriately arrange implementation timelines, allocate supporting resources and improve the success rate of AI projects [16]. Furthermore, the continuous monitoring of nursing managers’ AI readiness can serve as an important indicator for evaluating the effectiveness of AI training programmes and tracking the development of organisational AI capabilities.

### 2.4. Research on AI Readiness Assessment Tools

With the rapid development of AI applications in the healthcare sector, the need to assess the AI readiness of healthcare professionals and organisations has become increasingly urgent. Recently, researchers have developed various AI readiness assessment tools; however, specialised scales targeting the nursing field, particularly the nursing manager population, remain relatively scarce.

Jöhnk et al. [17] identified key factors of organisational AI readiness through interviews and proposed an assessment framework encompassing dimensions such as strategic alignment, resource availability, data management and employee capabilities. However, this framework primarily targets corporate organisations and fails to adequately consider the unique characteristics of healthcare institutions. In the healthcare sector, some researchers have attempted to develop readiness assessment tools for specific AI application scenarios, such as acceptance scales for AI‐assisted diagnostic systems and readiness questionnaires for clinical decision support systems. However, these tools mostly focus on the physician population or specific technological applications without considering the unique characteristics of the nursing profession [18].

The Nursing Leaders’ Readiness for AI Integration Scale developed by Kotp et al. [16] fills an important gap in this field. Based on a systematic literature review and incorporation of the characteristics of nursing management practice, the scale provides an assessment framework encompassing three dimensions: leadership support, staff readiness and technological infrastructure. The scale comprises 20 items rated on a 4‐point Likert scale and demonstrated good psychometric properties in a sample of Egyptian nursing leaders (Cronbach’s *α* = 0.90, test–retest reliability = 0.854 and good confirmatory factor analysis [CFA] model fit). The development of this scale provides an effective tool for assessing nursing managers’ AI readiness and has significant theoretical and practical value.

Currently, although nursing managers’ AI readiness faces unique challenges in China, no relevant tools are available to assess it in the Chinese context. The Chinese healthcare system has distinctive organisational structures and management models that differ from those in Western countries in terms of nursing managers’ role positioning, scope of responsibilities and decision‐making authority. Furthermore, Chinese nursing personnel’s educational backgrounds, career development pathways and attitudes towards new technologies may differ from those in other countries. These factors suggest that directly applying AI readiness scales developed abroad may not accurately reflect the readiness of Chinese nursing managers. Therefore, a systematic cross‐cultural adaptation of existing scales is urgently needed to ensure their applicability and validity in the Chinese nursing manager population.

## 3. The Study

### 3.1. Aim

The main objectives of this study were as follows. First, we translated and adapted the Nurse Leaders’ Readiness for Artificial Intelligence Scale (NLRAI) into Chinese following international standardised procedures. Second, we examined the reliability of the Chinese version of the scale, including its internal consistency and split‐half reliability. Third, we verified the construct, convergent, discriminant and content validity of the scale. Furthermore, we examined the psychometric properties of the scale among Chinese nurse leaders and explored the status and influencing factors of AI readiness in this population.

### 3.2. Design

This multinational cross‐sectional study aimed to cross‐culturally validate the Nursing Leaders’ Readiness for AI Integration Scale.

### 3.3. Translation and Cognitive Interviews

#### 3.3.1. Translation and Back‐Translation

With permission from Professor Mohamed Hashem Kotp, the original developer of the NLRAI, a research team was formed to perform translation and back‐translation based on the Brislin model. For forward translation, two nurses with nursing education backgrounds and overseas study experience independently translated the scale. For translation synthesis, the research team compared both translations, discussed the differences, and then revised and merged them into a single Chinese version. For back‐translation, two bilingual translators who had completed their graduate nursing education in English‐speaking countries independently translated the Chinese version back into English. Finally, for the back‐translation review, the research team reviewed and corrected the back‐translated version after completing the translation.

#### 3.3.2. Expert Enquiry

In November 2025, we conducted two rounds of email‐based expert consultations on cultural adaptation and a review of the translated scale. Experts were selected based on the following criteria: (1) holding a master’s degree or higher or a senior professional title, (2) at least 5 years of work experience in nursing management or nursing education, (3) at least 3 years of involvement in nursing informatics and (4) voluntary participation in this study. Twelve experts assessed the preliminary Chinese version of the NLRAI based on semantic accuracy, language expression and conceptual consistency. A 4‐point scale was used to rate the relevance of each item to the theme: 1 = not relevant, 2 = somewhat relevant, 3 = quite relevant and 4 = highly relevant. The content validity index (CVI) was calculated using expert ratings. The items were then revised based on expert suggestions to improve the language clarity and cultural suitability of the Chinese version. The reliability of the expert consultations was evaluated using expert positive and authority coefficients [19].

#### 3.3.3. Cognitive Interviews and Presurvey

The questionnaire adopted the scoring method used for the original scale. Before the formal survey, we used purposive sampling to select 10 nursing managers from tertiary Grade A hospitals in Zhengzhou, Henan Province, China. This pretest aimed to verify whether the adapted version fit the local cultural context and the translated items were clear and easy to understand. The researchers asked whether any items were difficult to understand or answer or had unclear expressions.

### 3.4. Psychometric Evaluation

The psychometric properties of the 20‐item Nursing Leaders’ Readiness for AI Integration Scale were tested, including four types of validity (construct, convergent, discriminant and content validity) and two types of reliability (internal consistency and split‐half reliability).

#### 3.4.1. Sample and Data Collection

From October 2025 to January 2026, a survey was conducted in seven hospitals in China using purposive sampling. Participants were nursing managers in healthcare institutions, including vice presidents in charge of nursing, nursing department directors, nursing department deputy directors, nursing department officers, head nurses, teaching secretaries, research secretaries and quality control nurses.

Inclusion criteria were as follows: (1) nurses who held a formal nursing leadership or management position at a healthcare institution during the study period and had at least 6 months (determine the appropriate duration) of work experience in their current leadership or management role; (2) able to independently understand and complete the questionnaire, voluntarily participate in the study and sign an informed consent form.

Exclusion criteria were as follows: (1) participants who, during the study period, were on extended leave, seconded, or rotated to positions outside of nursing management; (2) participants who had previously been involved in the translation and back‐translation of the ‘Nursing Leaders’ AI Readiness Scale’, expert consultation, cognitive interviews or pilot surveys.

According to the factor analysis requirements, the sample size should be 5–10 times the number of items [20], and the sample size for CFA should be larger than that for exploratory factor analysis (EFA). The NLRAI contains 20 items in total, and considering 20% invalid questionnaires, the estimated required sample size was 125–250. This study was conducted in two stages. In the first stage, 256 valid questionnaires collected in October 2025 were used for item and EFAs. In the second stage, after completing the statistical analysis, the revised questionnaire was officially distributed in November 2025. A total of 330 valid responses from this period were used for CFA and reliability testing. Data were collected until January 2026, resulting in 762 valid responses. These data were used to examine the current level and influencing factors of AI readiness among nursing leaders. The researchers provided participants with information about the study, including the research purpose, completion methods, and the right to refuse or withdraw at any time. Participants provided signed informed consent before answering the questionnaire, which was distributed electronically.

#### 3.4.2. Measures

Sociodemographic data included participants’ gender, age, marital status, educational level, years of work experience, professional title, position, hospital type and level, whether they had participated in AI/nursing informatics–related training, and whether they had used AI tools or systems. Nursing leaders’ AI readiness was measured using the 20‐item Nursing Leaders’ Readiness for AI Scale.

#### 3.4.3. Items Analysis

For item analysis, this study used the critical ratio method, corrected item–total correlation and Cronbach’s α if item deleted. For the critical ratio method, total scores were calculated and ranked in descending order. Participants in the top 27% were assigned to the high‐scoring group, whereas those in the bottom 27% were assigned to the low‐scoring group. Independent samples *t*‐tests were performed to compare the two groups. Items with an absolute *t*‐value < 3 or *p* > 0.05 were considered for deletion. Corrected item–total correlations were calculated to assess the association between each item and the overall scale score after excluding the item itself. Items with a corrected item–total correlation < 0.40 were considered for deletion. In addition, Cronbach’s α if item deleted was calculated for each item. If deleting an item increased the overall Cronbach’s α coefficient, the item was considered potentially inconsistent with the overall construct and was further evaluated for deletion in combination with content relevance.

#### 3.4.4. Reliability Assessment

Cronbach’s α coefficient is a commonly used indicator for evaluating internal consistency reliability, with Cronbach’s α coefficient ≥ 0.70 generally indicating good internal consistency [21]. To assess split‐half reliability, the items were divided into two parts according to odd and even numbers, and the correlation coefficient between the scores of both parts was calculated. The closer the correlation coefficient is to 1, the higher the internal consistency of the scale.

#### 3.4.5. Validity Assessment

Construct validity was used to examine whether the scale had the expected theoretical structure and whether its internal components matched the theoretical framework. EFA and CFA were the main evaluation methods used [22]. EFA was performed using SPSS 27.0. Data were considered suitable for factor analysis when the KMO value was above 0.8, and Bartlett’s test of sphericity was statistically significant (*p* < 0.05). Principal component analysis with varimax rotation was applied to extract factors with eigenvalues greater than 1.0. Items with factor loadings above 0.45 were retained. CFA was conducted using AMOS 28.0 software. The maximum likelihood method was used for CFA. Model fit was assessed using the following five indices: *χ* ^2^/df, RMSEA, CFI, TLI and IFI. A good model fit was indicated by *χ* ^2^/df < 3, RMSEA < 0.08 and CFI, NFI and TLI all above 0.9 [23]. Convergent validity measures how strongly items within the same dimension correlate and reflects the internal consistency of the latent variable structure. This was assessed by calculating the average variance extracted (AVE) and composite reliability (CR). Higher AVE and CR values indicate stronger convergent validity. Generally, the CR and AVE values should exceed 0.70 and 0.50, respectively [24]. Discriminant validity was confirmed when the absolute correlation coefficient between dimensions was smaller than the square root of the AVE [25]. CVI evaluates how well the scale content matches what it intends to measure and includes three indicators: item‐level CVI (I‐CVI), scale‐level CVI based on universal agreement (S‐CVI/UA) and scale‐level CVI based on average (S‐CVI/Ave). Good content validity is indicated when I‐CVI ≥ 0.78, S‐CVI/UA ≥ 0.80 and S‐CVI/Ave ≥ 0.90 [26].

#### 3.4.6. Data Analysis

Data were analysed using SPSS 27.0 and AMOS 28.0. Categorical data were described using frequencies and percentages. Continuous data conforming to a normal distribution were described using mean ± standard deviation, and independent samples *t*‐tests and one‐way analysis of variance (ANOVA) were used for between‐group comparisons. Statistical significance was set at *p* < 0.05.

### 3.5. Ethical Considerations

This study adhered to the principles of confidentiality, beneficence and voluntariness. All participants could independently choose whether to participate and provided signed informed consent. This study was approved by the Research Ethics Board of Henan Provincial People’s Hospital on 31 May 2024.

## 4. Results

### 4.1. Expert Consultation Results

Twelve experts participated in two rounds of consultations. All 12 experts responded (effective response rate: 100%), indicating a high level of expert enthusiasm. The expert authority coefficients for the two rounds of consultation were 0.770 and 0.790, respectively, indicating a high level of expert authority. Following the first round of expert consultation, based on expert opinions, the translations of certain terms were modified and refined, such as changing ‘staff’ to ‘the staff of your organisation’ and ‘leadership’ to ‘the leadership of your organisation’. No further modifications were made following the second round of expert consultation. All scale items were ultimately retained.

### 4.2. Cognitive Interview and Presurvey Results

The presurvey results revealed that the time range for participants to complete the questionnaire was 6–10 min (7.8 ± 1.9 min), which is an appropriate length. None of the participants in the presurvey objected to the dimensions, items or questioning style of the scale, indicating a high level of acceptance.

### 4.3. Item Analysis

In October 2025, 256 nursing managers were recruited for item screening. The results of the critical ratio method showed t‐values ranging from 7.625 to 16.102, with all Ps < 0.001, indicating good discrimination of all items. The corrected item–total correlations ranged from 0.488 to 0.774, exceeding the recommended threshold of 0.40. The overall Cronbach’s α coefficient of the preliminary scale was 0.944, and Cronbach’s α if an item was deleted ranged from 0.939 to 0.943, all of which were lower than the overall Cronbach’s α coefficient. These findings indicated that no item reduced the internal consistency of the scale. Therefore, all 20 items were retained.

EFA was conducted on the 20 items. The KMO test value was 0.931, and Bartlett’s test of sphericity *χ* ^2^ value was 4837.152 (*p* < 0.001), indicating the suitability of the data for factor analysis. Using principal component analysis and varimax orthogonal rotation, three common factors with eigenvalues > 1 were extracted, with a cumulative variance contribution rate of 75.416%. Factor loadings of all items on their corresponding common factors were > 0.450, and item attribution was consistent with the original scale. Therefore, all items were retained. Table 1 presents the rotated factor loadings.

**TABLE 1:** Factor analysis (*n* = 256).

Items	Factor 1	Factor 2	Factor 3
Item 18	0.890	0.113	0.184
Item 17	0.885	0.176	0.184
Item 14	0.885	0.173	0.160
Item 16	0.864	0.223	0.216
Item 12	0.861	0.205	0.197
Item 15	0.858	0.206	0.221
Item 13	0.824	0.236	0.198
Item 3	0.120	0.880	0.108
Item 4	0.122	0.856	0.180
Item 5	0.110	0.811	0.178
Item 2	0.175	0.809	0.178
Item 6	0.278	0.730	0.299
Item 20	0.191	0.685	0.162
Item 1	0.286	0.676	0.166
Item 10	0.229	0.174	0.875
Item 8	0.206	0.232	0.855
Item 11	0.110	0.217	0.836
Item 7	0.212	0.232	0.816
Item 9	0.214	0.287	0.797
Item 19	0.200	0.058	0.701
Eigenvalue	5.766	4.813	4.504
Cumulative variance contribution rate (%)	28.83	52.894	75.416

### 4.4. Reliability Assessment

In November 2025, 330 nursing managers were recruited for the psychometric evaluation of the scale. Cronbach’s α coefficient was 0.824 for the overall scale and 0.915, 0.938 and 0.950 for each dimension, indicating that the overall scale and all dimensions had good internal consistency. The split‐half reliability of the scale was 0.916, further verifying the stability of the scale structure and consistency of the measurement results.

### 4.5. Validity Assessment

**FIGURE 1:**
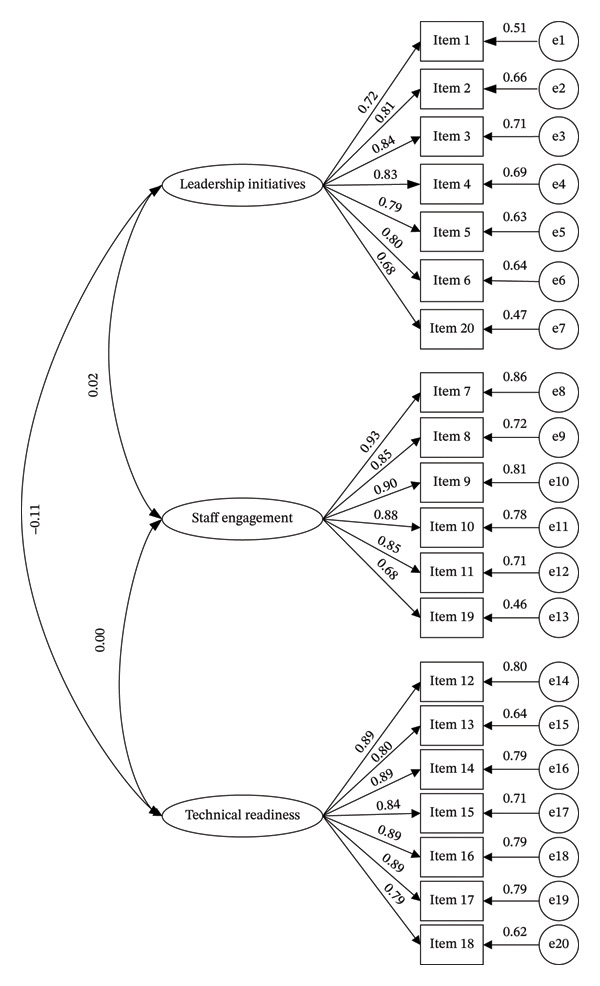
Three‐factor measurement model with standardized estimates. Oval nodes represent latent variables, rectangular nodes represent observed variables, and circular nodes represent error terms. Values beside the arrows from the latent variables to the observed variables indicate standardized factor loadings; values above the observed‐variable boxes indicate squared multiple correlations (*R* ^2^); and values beside the curved double‐headed arrows indicate correlations between the latent variables.

Regarding construct validity, based on the EFA results, a three‐factor first‐order model was constructed, and parameter estimation was performed using the maximum likelihood method. The results showed that the three‐factor model had a good fit: *χ* ^2^/df = 2.411, RMSEA = 0.065, CFI = 0.957, TLI = 0.951 and NFI = 0.929. The model is shown in Figure 1. Regarding convergent and discriminant validity, the standardised factor loadings of each item on its corresponding dimension ranged from 0.683 to 0.929, indicating that each item contributed greatly to its corresponding dimension. The AVE values for the three dimensions were 0.6146, 0.7244 and 0.7328, respectively (all > 0.5), and the CR values were 0.9174, 0.9399 and 0.9504, respectively (all > 0.70), indicating good convergent validity. The square roots of the AVE for each dimension were 0.784, 0.851 and 0.856, respectively; the absolute values of the correlation coefficients between the dimensions were 0.019, 0.115 and 0.004, respectively, all of which were less than the square root of the AVE, indicating that the scale had good discriminant validity (Table 2). Regarding content validity, the I‐CVI of the Chinese version of the Nursing Leaders’ Readiness for AI Scale ranged from 0.917 to 1.000, the S‐CVI/UA was 0.900 and the S‐CVI/Ave was 0.992. These results indicated that the scale has good content validity.

**TABLE 2:** Discriminant validity test results for each dimension.

Variable	Leadership strategic support dimension	Staff AI readiness dimension	Technology implementation capability dimension
Leadership strategic support dimension	0.784		
Staff AI readiness dimension	0.019	0.851	
Technology implementation capability dimension	−0.115	−0.004	0.856

### 4.6. Status and Influencing Factors of Nursing Leaders’ AI Readiness

A total of 766 questionnaires were collected, of which 762 were valid. The mean AI readiness score of nursing managers was 65.27 ± 9.78. Among the participants, 624 (81.89%) scored above 60 points, 136 (17.85%) scored between 40 and 60 points, and 2 (0.26%) scored below 40 points. Significant differences in AI readiness scores among nursing managers were found in terms of hospital type, hospital level, department, whether they had participated in AI/nursing informatics–related training, whether they had used AI tools or systems, and the frequency of AI tool use. Nursing managers in general hospitals scored higher than those in specialised hospitals, tertiary hospitals scored higher than secondary hospitals, nursing managers working in internal medicine departments had significantly higher AI readiness scores than those in other departments, those who had participated in AI/nursing informatics–related training scored higher than those who had not, those who had used AI tools or systems scored higher than those who had not, and nursing managers who used AI tools almost daily or 3–5 times per week scored higher than those with other frequencies of AI tool use. Table 3 presents the general characteristics of the survey respondents and the results of the analysis of the factors influencing AI readiness.

**TABLE 3:** General participant characteristics and results of the analysis of factors influencing AI readiness (*n* = 762).

Variables		Number (*n*, %)	Score ($\overset{¯}{x}\pm s$)	Test statistics	*p*
Sex	Male	27 (3.5)	65.74 ± 9.964	0.254	0.799
	Female	735 (96.5)	65.25 ± 9.788		

Marital status	Married	635 (83.3)	65.18 ± 9.559	−0.563	0.574
	Widowed/divorce/unmarried/other	127 (16.7)	65.72 ± 10.892		

Educational level	College degree and below	46 (6.0)	62.52 ± 10.568	2.100	0.123
	Bachelor’s degree	645 (84.6)	65.52 ± 9.632		
	Postgraduate degree or above	71 (9.3)	64.82 ± 9.788		

Hospital category	Comprehensive hospital	744 (97.6)	65.37 ± 9.803	2.125	**0.048**
	Specialised hospital	18 (2.4)	61.11 ± 8.366		

Hospital ownership	Public hospital	758 (99.5)	65.29 ± 9.803	0.772	0.440
	Private hospital	4 (0.5)	61.50 ± 5.972		

Hospital level	Secondary hospital	154 (20.2)	61.68 ± 8.283	−5.768	**< 0.001**
	Tertiary hospital	608 (79.8)	66.18 ± 9.936		

Professional title	Junior	184 (24.1)	66.39 ± 10.477	2.497	0.083
	Intermediate	470 (61.7)	65.18 ± 9.608		
	Senior	108 (14.2)	63.77 ± 9.193		

Department	Nursing department	14 (1.8)	63.57 ± 10.711	3.230	**0.002**
	Medical department	241 (31.6)	67.19 ± 9.960		
	Surgical department	220 (28.9)	65.37 ± 9.970		
	Department of obstetrics and gynaecology	52 (6.8)	63.90 ± 9.668		
	Department of paediatrics	32 (4.2)	61.81 ± 6.837		
	Emergency and critical care units	63 (8.3)	65.05 ± 8.931		
	Operating room and department of anaesthesiology	31 (4.1)	61.06 ± 6.191		
	Others	109 (14.3)	64.05 ± 10.274		

Participation in AI/nursing informatics–related training	Yes	521 (68.4)	66.26 ± 9.729	4.182	**< 0.001**
	No	241 (31.6)	63.12 ± 9.588		

Use of AI tools or systems	Yes	671 (88.1)	65.70 ± 9.688	3.202	**0.002**
	No	91 (11.9)	62.13 ± 10.002		

Frequency of using AI tools	Daily	165 (24.6)	68.38 ± 9.897	8.254	**< 0.001**
	3 to 5 times weekly	182 (27.1)	66.85 ± 9.337		
	1 to 2 times weekly	156 (23.2)	64.22 ± 9.018		
	1 to 3 times monthly	71 (10.6)	64.21 ± 8.433		
	Rarely (less than once a month)	97 (14.5)	62.42 ± 10.450		

*Note:* Bold values indicate that the value is less than 0.05 and has statistical significance.

## 5. Discussion

### 5.1. Application Value of the NLRAI Scale

With the in‐depth application of AI technology in the healthcare field, AI readiness has become a key element of core competencies for nursing managers (Fennimore & Wolf, 2021). This study provided empirical support for the applicability of the AI readiness concept in the Chinese nursing management context. Through systematic cross‐cultural adaptation and psychometric testing, the validity of AI readiness as a multidimensional theoretical framework in the Chinese cultural context was verified, thus enriching the cross‐cultural research evidence for AI readiness. Furthermore, this study provides a methodological reference for other countries and regions to conduct similar cross‐cultural adaptation studies.

At the practical level, the Chinese version of the Nursing Leaders’ Readiness for AI Scale can provide healthcare institutions with a standardised tool for assessing nursing managers’ AI readiness status. Healthcare institutions can use this scale to systematically identify specific gaps among nursing managers in dimensions such as leadership support, staff readiness and technological infrastructure, thereby developing targeted training and development programmes [15].

Research has shown that organisational AI readiness levels directly affect the implementation effectiveness and success rate of AI projects [17]. This scale can provide data support for the development of AI project implementation strategies. Before initiating AI projects, healthcare institutions can use this scale to assess the overall readiness level of nursing management teams and identify potential barriers and challenges that may be encountered during implementation, thereby developing more practical and feasible implementation plans, rationally allocating support resources and reducing the risk of project failure.

### 5.2. Reliability of the Assessment Scale

This study adopted a standardised translation process. Through team‐based translation, back‐translation and two rounds of expert consultation, the Chinese version of the Nursing Leaders’ Readiness for AI Scale was developed, ensuring professional quality and cross‐cultural suitability. The effective response rate, expert authority coefficient and Kendall’s concordance coefficient indicated that the experts had high levels of participation, expertise and agreement. Scale reliability was assessed using internal consistency and split‐half methods. Cronbach’s α was 0.824 for the overall scale and 0.915, 0.938 and 0.950 for the three dimensions. The split‐half reliability was 0.916. All values exceeded the threshold of 0.7. In summary, the Chinese version of the scale showed satisfactory psychometric properties in terms of internal consistency and structure.

### 5.3. Validity of the Assessment Scale

This study evaluated the validity of the Chinese version of the Nursing Leaders’ Readiness for AI Scale based on four aspects: construct, convergent, discriminant and content. For construct validity, EFA was first used to identify the underlying factor structure. Three factors with eigenvalues above 1 were extracted, explaining 75.416% of the total variance, meeting the recommended criteria. The factor loadings were above the 0.45 cutoff for all items, ranging from 0.676 to 0.890. This indicated strong relationships between items and their factors, suggesting a stable structure. Based on these findings, a hypothesised model was built and tested using CFA with 330 samples. The model fit indices were as follows: *χ* ^2^/df = 2.411, RMSEA = 0.065, CFI = 0.957, TLI = 0.951 and NFI = 0.929. All values met the acceptable thresholds, confirming that the three‐factor model fit well and supported the scale’s theoretical structure. Regarding convergent validity, the AVE values for the three dimensions were all above 0.50, at 0.6146, 0.7244 and 0.7328. This indicated that each factor explained a large proportion of the variance in its items. The CR values were 0.9174, 0.9399 and 0.9504, all of which exceeded 0.70, indicating stable internal structure and high measurement consistency for each dimension. Thus, the scale demonstrated satisfactory convergent validity and CR. For discriminant validity, the absolute correlation coefficients between dimensions were 0.019, 0.115 and 0.004, all of which were lower than the square root of the AVE values. This indicated that the dimensions were distinct from each other. Content validity was assessed using the Delphi method for expert evaluation. The results showed that the I‐CVI ranged from 0.917 to 1.000, S‐CVI/UA was 0.900 and S‐CVI/Ave was 0.992. All values met or exceeded the recommended standards. Thus, the Chinese version of the scale showed good psychometric properties across all four validity dimensions.

### 5.4. Status and Influencing Factors of Chinese Nursing Managers’ AI Readiness

The mean AI readiness score of Chinese nursing managers was 65.27 ± 9.78, with 81.89% of participants scoring above 60 points, indicating that most nursing managers held a positive attitude towards AI technology integration and possessed a good state of AI readiness. These results are consistent with the policy context of China’s active promotion of digital transformation and smart hospital construction in the healthcare field [27]. Compared with the findings of Kotp et al.[16] among Egyptian nursing leaders, the overall AI readiness level of Chinese nursing managers in this study was slightly higher. Kotp et al.[16] showed that 36.8% of Egyptian nursing leaders had a high level of AI readiness, 34.0% had a moderate level and 29.2% had a low level. This difference may be related to the different levels of healthcare informatisation development, degree of AI technology popularisation and opportunities for nursing managers to access new technologies between the two countries. Evidence from Turkey provides a broader cross‐cultural perspective. Erkayıran and Aslan [31] found that Turkish nurses were generally optimistic about the potential benefits of AI in healthcare, but their knowledge of AI remained limited, and concerns regarding patient privacy and ethical issues were important barriers to AI integration. Although the Turkish study focused on practicing nurses rather than nursing managers, its findings suggest a similar pattern in which positive attitudes towards AI do not necessarily translate into sufficient readiness for implementation. The relatively favourable readiness profile observed among Chinese nursing managers in the present study may be associated with their managerial roles, greater exposure to institutional digital transformation, AI‐related training opportunities, hospital digital health infrastructure and the policy environment supporting smart hospital development in China. The recent rapid development of medical AI in China has provided nursing managers with opportunities to access and understand AI technologies. The National Health Commission of China has successively issued a series of policy documents to promote the development of healthcare informatisation and intelligentisation, creating a favourable policy environment for the application of AI technology in the healthcare field. These factors may have contributed to improvements in participants’ AI readiness levels. However, 17.85% of nursing managers still scored at a moderate level between 40 and 60 points, and 0.26% of nursing managers scored below 40 points, indicating that some still have deficiencies in AI readiness. This suggests that when promoting AI technology application, healthcare institutions must consider the special needs of nursing managers and provide targeted support to narrow the readiness gap within this group [5].

This study found that nursing managers in general hospitals had higher AI readiness scores than those in specialised hospitals, while nursing managers in tertiary hospitals scored higher than those in secondary hospitals. These results may be related to differences in resource allocation, technological infrastructure and organisational support among different types and levels of healthcare institutions. General hospitals are typically larger, have comprehensive department settings, and cover a wide range of diseases, and thus face more complex and diverse clinical decision‐making scenarios. These conditions may create a more urgent need for AI‐assisted decision‐making, and therefore, they may be more proactive in introducing and applying AI technology [32]. As regional medical centres, tertiary hospitals undertake important functions such as diagnosing and treating difficult and critical cases, providing medical education and driving scientific research innovation, and usually have higher sensitivity and acceptance of new technologies [6]. The advantages of tertiary hospitals in informatisation infrastructure, technical talent and research resources can provide better organisational support for the introduction and application of AI technology, thereby enhancing nursing managers’ awareness of and readiness for AI technology [2]. These results suggest that when promoting the application of AI technology in nursing, attention should be paid to the digital divide between different types and levels of healthcare institutions. Policymakers and healthcare administrators should increase support for specialised hospitals and primary healthcare institutions, thereby helping nursing managers in these institutions improve their AI readiness through resource allocation, technical assistance and training support to promote the balanced development of AI technology [30].

Furthermore, internal medicine encompasses a wide variety of diseases, and the care needs for chronic disease management and patients with multiple comorbidities are complex, resulting in a high demand for AI‐assisted decision‐making tools based on big data analysis and predictive models [8]. Nursing managers in internal medicine departments may encounter and use AI tools more frequently in their daily work, thereby accumulating more experience in AI application and demonstrating higher AI readiness.

Nursing managers who had participated in AI‐ or nursing informatics–related training had significantly higher AI readiness scores than those who had not. This finding is consistent with those of previous research, emphasising the critical role of education and training in enhancing AI readiness among healthcare professionals [4]. Training can help nursing managers systematically understand the basic principles, application scenarios and development trends of AI technology, eliminate unfamiliarity and fear of new technology, enhance awareness and confidence in AI technology and impart management knowledge and skills for AI project implementation, thereby helping nursing managers better understand how to integrate AI technology into nursing practice effectively [15]. At the managerial level, fostering digital literacy and specialised AI competencies requires a structured approach. International evidence underscores the urgent need to integrate AI literacy into nursing education, enabling nursing managers to develop both the technical competencies and ethical decision‐making skills needed to guide AI adoption [31]. Nursing workforce competencies have also been identified as critical mediators between AI utilisation and productivity outcomes, further highlighting the importance of targeted competency development at the managerial level [15]. Therefore, policymakers and healthcare organisations should consider incorporating AI and informatics training into the continuing professional development of nursing managers and establishing standardised AI competency frameworks to systematically cultivate the digital literacy and specialised AI capabilities required for effective leadership in intelligent healthcare.

In terms of AI tool usage experience, nursing managers who had used AI tools or systems had significantly higher AI readiness scores than those who had not. Furthermore, nursing managers who used AI tools almost daily or 3–5 times per week scored higher than those who used them less frequently. These results indicate that practical usage experience is an important factor influencing nursing managers’ AI readiness. Through the actual use of AI tools, nursing managers can intuitively experience the functions and value of AI technology and understand its application potential in nursing practice, thereby enhancing their confidence in and positive attitudes towards AI technology. Conversely, nursing managers who rarely or never use AI tools may feel unfamiliar with AI technology, lack direct usage experience and demonstrate more uncertainty and concern when facing AI‐related decisions [13]. These findings suggest that when promoting AI technology application, healthcare institutions should create opportunities for nursing managers to access and use AI tools and may consider piloting AI applications in low‐risk scenarios first, allowing nursing managers to gradually become familiar with AI technology through practice, accumulate usage experience, and build application confidence. Nursing managers should be encouraged to integrate AI tools into their daily workflows and increase their usage frequency to enhance their AI readiness and application capabilities.

Moreover, because attitudes towards AI, communication patterns, professional roles and trust in technology may vary across cultural and organisational contexts, healthcare institutions should avoid relying on a single standardised implementation approach. Instead, they should develop culturally responsive AI adoption strategies by assessing local staff needs, technology acceptance, ethical concerns, workflow characteristics and regulatory requirements before implementation. International evidence suggests that successful translation of AI into healthcare practice depends not only on technical performance, but also on contextual readiness, stakeholder engagement, governance structures and implementation processes [32, 33]. From a nursing management perspective, institutions can establish multidisciplinary AI implementation teams, involve nursing managers from diverse cultural and professional backgrounds in decision‐making, provide locally adapted AI training and use feedback from pilot implementation to refine adoption strategies. These approaches may help healthcare organisations translate the present findings into cross‐cultural strategies for AI adoption and support the more equitable, acceptable and sustainable integration of AI into nursing management practice [8, 34].

## 6. Study Limitations

The sample in this study was mainly from central China, and regional differences in economic and technological development may have affected the survey results. Although this study followed standardised cross‐cultural adaptation procedures, owing to differences among countries in healthcare systems, organisational cultures and technological development levels, the cultural adaptability of some items still needs further validation in broader applications. Furthermore, considering the rapid development of AI technology, nursing managers’ cognition and readiness status for AI may change with technological advancements and the accumulation of application experience. Future studies could adopt longitudinal designs to track the trajectory of changes in nursing managers’ AI readiness and its influencing factors.

## 7. Conclusion

This study conducted a cross‐cultural adaptation of the Chinese version of the Nursing Leaders’ Readiness for AI Scale according to international standardised procedures and systematically examined its psychometric properties among Chinese nursing managers. The findings showed that the Chinese version of the scale has satisfactory reliability and validity. Thus, it can be used as a valid tool to assess AI readiness among Chinese nursing managers. However, because this scale is newly introduced in China, the sample representativeness may be limited. Future studies should use this scale with larger samples to conduct more extensive surveys. By understanding the distribution characteristics and influencing factors of AI readiness among nursing managers in different regions and healthcare institutions at different levels, empirical evidence can be provided for the formulation of nursing informatics policies at the regional or national level. This study revealed the overall status of AI readiness among Chinese nursing managers and its influencing factors. Hospital type, hospital level, department, training experience, AI tool usage experience and usage frequency significantly affected nursing managers’ AI readiness. These findings provide important evidence for the development of strategies to enhance nursing managers’ AI readiness.

## Funding

This study was supported by the Henan Provincial Key Laboratory of Nursing Medicine Fund (HNSYHLKT202409) and Henan Province Medical Science and Technology Research Project (LHGJ20250051).

## Ethics Statement

This survey was approved by the Henan Provincial People’s Hospital Ethics Review Committee (ID number: 2024‐114). Any data used in the submitted manuscript were legally obtained.

## Conflicts of Interest

The authors declare no conflicts of interest.

## Data Availability

The data that support the findings of this study are available from the corresponding author upon reasonable request.
